# News and Coming Events

**Published:** 1909-07-03

**Authors:** 


### News and Coming Events.

At the annual general meeting of the London Nottinghamshire Society, held on June 24, Mr. Sydney Stephenson, M.B., F.R.C.S., was elected President of the Society for 1909-1910. Mr. Stephenson has been for some time past editor of *The Ophthalmoscope*.

The Raymond Horton-Smith Prize in the University of Cambridge for 1909 for the best Thesis during the past year for the Degree of Doctor of Medicine has been awarded to Mr. Henry Hallett Dale, M.A., M.B., B.C., of Trinity College. Mr. Cecil Herbert Winter Page, M.A., of Corpus Christi College, was adjudged *Proxime accessit*.

The therapeutical and pharmacological section of the Royal Society of Medicine will meet at Cambridge on July 10, at 3 p.m.; there will be demonstrations in the Pharmacological Laboratory by Professor W. E. Dixon and others, and at 6.30 p.m. the dinner will be held in the Hall of Downing College.

The governors and medical staff of Guy's Hospital will give a garden party on Thursday, July 8, at 3.15 p.m., when the medals and prizes for the year will be distributed to the successful students by his Grace the Duke of Devonshire. The laboratories, museums, college, the Henriette Raphael Nurses' Home, and wards will be open to inspection from 3 to 5.30 p.m. Music will be provided in the grounds. Academic costume will be worn.

Dr. James Douglas, of Quebec and New York, has founded the Douglas Studentship for research in Actino-Therapeutics, with special reference to cancer, at Guy's Hospital Medical School. The studentship is tenable for one year, is of the value of £300, and is open to all persons who possess a registrable British medical qualification. The student will be required, unless otherwise permitted by the Treasurer, to carry on his investigations at Guy's Hospital, under the supervision of the surgeon to the Actino-Therapeutic Department.

We regret to announce the death, which occurred last week at Edinburgh, in his sixtieth year, of Professor Daniel John Cunningham, M.D., F.R.S., LL.D., D.C.L., D.Sc., etc., Professor of Anatomy in the University of that city, and Dean of the Medical Faculty. Professor Cunningham's name and works are well known to all British students and practitioners of medicine. His father was Principal of St. Mary's College, St. Andrews, and he himself proceeded from Crieff Academy to Edinburgh University, where he graduated M.B. in 1874 and M.D. in 1876. After holding the post of Demonstrator of Anatomy in that University he was appointed to the Chair of Anatomy and Surgery at Dublin University, which he vacated in 1903, after 20 years' excellent service, to become Professor of Anatomy in his old University. Professor Cunningham, among his many appointments, had held the post of Examiner in Anatomy to the Universities of Oxford, Cambridge, Edinburgh, London, and Victoria, and in examinations for the Services as well as that of President of the Royal Zoological Society of Ireland and Vice-President of the Royal Dublin Society. He was a member of the South African Hospitals Commission and the Vice-Regal Commission on the Inland Fisheries of Ireland. His most important literary activities were the authorship of his exceedingly popular and useful Manual of Practical Anatomy; the editorship of the valuable Textbook of Anatomy which bears his name; and the acting editorship of the *Journal of Anatomy and Physiology*.

The Chesterfield Medal in Dermatology, issued by St. John's Hospital for Diseases of the Skin, Leicester Square, has this year been awarded to Dr. C. A. McBride, M.D., L.R.C.P.

News has been received from the Sleeping Sickness Camp, near Kisii, British East Africa, of the death, from malaria, of Dr. Henry Hugh Baker, at the age of 31. Dr. Baker was educated at University College, Oxford, and St. Mary's Hospital, London, and qualified in 1905. He was Government Medical Officer at Nairobi, British East Africa, and had previously been Medical Officer at Kharga Oasis, West Egypt.

The annual meeting of the Poor Law Medical Officers' Association will take place on Tuesday, July 6, at the Guildhall, London, when a conference of the Poor Law medical officers of England and Wales, to consider the recommendations of the Royal Commission on Poor Law Medical Relief, which will be opened by the Lord Mayor in the Council Chamber at 11 a.m. Papers will be read by Dr. Major Greenwood, D.P.H.; Mr. C. S. Loch, Secretary of the London Charity Organisation Society; Mrs. Sidney Webb; Dr. F. S. Toogood, Medical Superintendent, Lewisham Infirmary; and Dr. G. F. McCleary, M.O.H., Hampstead. The papers will be followed by discussions. The annual dinner will be held at the Waldorf Hotel, Aldwych, London, at 7.30 p.m. on the same evening, when Surgeon-General Evatt, C.B., President of the Association, will preside. The price of tickets is 7s. 6d., exclusive of wine. Poor-law medical officers wishing to be present should communicate with the Hon. Secretary, Dr. Major Greenwood, 243 Hackney Road, N.E. Ladies are admissible as guests, as well as to afternoon tea at the Mansion House from 3.30 to 6 p.m.

On June 24, the Festival of St. John Baptist, the members and associates of the Order of St. John of Jerusalem in England held their annual commemoration service at St. John's Parish Church, Clerkenwell, the anniversary sermon being preached by Bishop Ormsby, Sub-Prelate of the Order. Afterwards the General Assembly was held in the Chapter Hall at St. John's Gate. Reports were received from the Committee of the British Ophthalmic Hospital, Jerusalem, belonging to the Order, and from the Committee of the Ambulance Department.

